# Factors Influencing Nutritional Intake and Interests in Educational Content of Athletes and Sport Professionals Toward the Development of a Clinician-Supported Mobile App to Combat Relative Energy Deficiency in Sport: Formative Research and a Description of App Functions

**DOI:** 10.2196/45098

**Published:** 2023-07-26

**Authors:** Jacob T Mey, Christine A Karpinski, Shengping Yang, Joseph D Madere, Tavis Piattoly, Ronnie Harper, John P Kirwan

**Affiliations:** 1 Pennington Biomedical Research Center Baton Rouge, LA United States; 2 West Chester University West Chester, PA United States; 3 My Sports Dietitian, LLC Baton Rouge, LA United States

**Keywords:** dietitian, malnutrition, mHealth, mobile health, performance, RED-S, relative energy deficiency in sport, sports nutrition, technology

## Abstract

**Background:**

Relative energy deficiency in sport (RED-S) as a consequence of athlete malnutrition remains a prominent issue. However, it remains underrecognized, in part due to the perceived outward health of athletes. The Eat2Win app was designed to combat RED-S and athlete malnutrition by providing education, behavior modification, and direct communication with expert sports dietitians to athletes and sport professionals (professionals who work with athletes, eg, sport coaches and athletic trainers).

**Objective:**

The purpose of this formative research was to gain critical insight on motivators and barriers to optimal nutritional intake from both the athletes’ and sport professionals’ perspectives. Additionally, since these 2 groups represent the primary end users of an app aimed at improving athlete nutrition and reducing the risk of RED-S, a secondary objective was to gain insight on the preferences and perceptions of app-based educational content and functionality.

**Methods:**

An electronic survey was developed by an interdisciplinary team of experts. Survey questions were established based upon prevailing literature, professional dietetic field experience, and app design considerations to obtain respondent knowledge on key sports nutrition topics along with motivations and barriers to meal choices. Additionally, the survey included questions about the development of an integrative, clinician-support app aimed at addressing RED-S. These questions included preferences for educational content, modes of in-app information, and communication delivery for the target population (app end users: athletes and sport professionals). The survey was distributed through Research Electronic Data Capture (REDCap) to athletes and sport professionals using targeted email, social media, and community engagement campaigns. The electronic survey was available from May 4 to August 2, 2022.

**Results:**

Survey respondents (n=1352) included athletes and professionals who work with athletes from a variety of settings, like high school, collegiate, professional, and club sports. Respondents reported high interest in 8 core sports nutrition topics. The preferred modes of information and communication delivery were visual formats (eg, videos and infographics) and in-app alerts (eg, direct messaging and meal reminders). Only athlete respondents were asked about motivators and barriers that influence meal choices. “Health” and “sports performance” were the highest scoring motivators, while the highest scoring barriers were “cost of food,” “easy access to unhealthy food,” and “time to cook or prepare food.” Notably, survey respondents provided positive feedback and interest using a novel function of the app: real-time meal feedback through food photography.

**Conclusions:**

The Eat2Win app is designed to combat RED-S and athlete malnutrition. Results from this study provide critical information on end-user opinions and preferences and will be used to further develop the Eat2Win app. Future research will aim to determine whether the Eat2Win app can prevent RED-S and the risk of athlete malnutrition to improve both health and performance.

## Introduction

Athletes are an underrecognized population for malnutrition, which leads to profound negative health effects characterized in the relative energy deficiency in sport (RED-S) [1]. High school and collegiate athletes are especially vulnerable to RED-S. The negative health effects of RED-S include major hormonal and physical impairments like the Female Athlete Triad [2] (disordered eating, amenorrhea, and osteoporosis), with similar concerns in male athletes [3]. Despite robust efforts to improve high school and collegiate athlete nutrition [4], RED-S remains a major health concern [5]. It is critical to prevent RED-S early, as long-term consequences to muscle and bone health can persist throughout the life span. This can lead to an increased risk of injuries, low bone mineral density, and subsequently osteoporosis, fractures, and frailty later in life [6]. These are serious conditions that can create a vicious cycle of reduced independence and low quality of life [7].

RED-S is caused, in part, by suboptimal athlete nutritional intake. Critical factors that lead to suboptimal athlete nutritional intake include gaps in nutrition knowledge [8] and low exposure to expert sources of nutrition information (eg, geographic, economic, or awareness limitations that prevent access to expert nutrition services) [9]. Improving nutrition education and exposure to nutrition experts has a positive impact on athlete nutritional intake. Various forms of sports nutrition education in all levels of athletics have been proposed [10,11] and are effective when implemented [12,13]. When a nutrition expert (eg, sports dietitian) is integrated within university sports teams, nutritional intake substantially improves [14]. Despite the success of these approaches, access to nutrition education and nutrition experts remains a critical barrier for athletes. Accordingly, surveys of both athletes and sport professionals (professionals that work with athletes; eg, coaches and athletic trainers) show that they frequently score low on evidence-based nutrition knowledge assessments and report barriers to accessing expert nutrition professionals [8,15-18]. One solution to address these critical barriers and improve athlete nutritional intake is the use of mobile apps. Mobile apps overcome barriers to access and offer an opportunity to connect athletes and sport professionals directly to evidence-based nutrition information and provide direct exposure to expert sports dietitians.

Therefore, a novel mobile app, Eat2Win, was developed by an expert team of sports nutrition professionals with app functions designed to overcome the critical barriers to improving athlete nutritional intake. The Eat2Win app connects athletes and sport professionals directly with expert registered dietitians in addition to its novel in-app food photography platform that allows for real-time feedback on meal choices and nutrition education. Here, we present formative research on a population of athletes and sport professionals regarding preferences for nutrition educational content, modes of in-app information and communication delivery, motivations and barriers that influence meal choices, and opinions on the Eat2Win app’s functions. We also present a description of current app functionality aimed at addressing these barriers, which may support the use of the Eat2Win app in future research for optimizing athlete nutritional intake. These data will be used to inform additional app developments to target the prevention of RED-S and athlete malnutrition.

## Methods

### RED-S, Athlete Malnutrition and Sport Nutrition Knowledge Literature Review

To establish the contemporary issues and knowledge gaps pertaining to athlete malnutrition and RED-S, we searched PubMed and Google Scholar with the search terms “relative energy deficiency in sport” with “knowledge,” “athlete,” and “trial.” Finally, we searched for “nutrition knowledge questionnaire,” “athletes,” and “malnutrition.” All articles fell within the years 2014 through 2022. References that were identified within primary research and topical review articles were considered for inclusion as well. Articles were restricted to those published in the English language. We also incorporated expert insight and professional opinions from practicing sports dietitians.

### Description of the Technology (Eat2Win App Features)

The Eat2Win mobile phone app is a resource that an athlete can download from the Apple and Google Play app stores. The goal of the Eat2Win app is to help athletes improve their eating habits and, as a result, their overall health and athletic performance. The app was built using the cross-platform mobile framework software developed by Google. Flutter (Google) is the main development platform, and Firebase (Google) is where the data is processed and stored. Both are cutting-edge platforms with a history of performance and data security. Flutter maintains the leading market share for all mobile apps in the Apple and Google Play app stores. The Eat2Win app was first published in January of 2018 and has gone through 3 major revisions in design and development with over 40,000 downloads.

The app provides athletes with a means to identify their unique caloric needs and offers them a wide variety of customized meal plan guides that meet their specific energy needs. Athletes can choose to set meal reminders as well as how they want their meals distributed. It is customized to each athlete’s personal needs and preferences. The app contains meal plan guides, which include breakfast, lunch, dinner, snacks, and postworkout meals. Athletes can also easily track and log their body weight, wellness, hydration, sleep, and meals. A photographic food log makes it easy for athletes to see their eating patterns over time through meal photos stored within the app. The meal photos are also used to provide real-time nutrition feedback through simple, actionable statements (see Table S1 in Multimedia Appendix 1).

Having web-based access with a highly qualified sports dietitian is a key feature of the app. Users can directly message (in-app) an assigned sports dietitian if they have sports nutrition-related questions, and if needed, upgrade to work 1-on-1 with that professional. A team approach is also available within the app, as “organization administrators” can personalize the experience for all their users by implementing gamification events with teams or groups of athletes. For example, as an athlete engages with the app features, they score points and compete against their peers, encouraging app use. Organization administrators are the professionals that oversee athletes, teams, or institutions; for example, coaches and athletic directors. Organization administrators can also distribute selected sports nutrition content to individuals or groups of athletes based on a series of short thematic videos based on need, for example, off-season nutrition and game-day nutrition. In addition, organization administrators can assign “nutrition monitors” to each athlete. A nutrition monitor is an individual who can see athletes’ profiles and activities within the app, for example, strength and conditioning specialists and position coaches. The nutrition monitor’s goal is to provide encouragement and accountability to their assigned athletes. When athletes communicate with their assigned sports dietitian, their assigned nutrition monitors are maintained in the communication loop as well. Through this engagement and communication platform, the Eat2Win app is designed to deliver evidence-based nutrition information and direct interaction with sports nutrition experts. This survey will inform developments of the current content and platform to improve the Eat2Win app and overcome barriers to optimal nutritional intake for athletes.

### Eat2Win Survey Development and Implementation

A formative research survey on athlete nutrition and the Eat2Win app functions was developed by an interdisciplinary team of experts at My Sports Dietitian, a small business in Louisiana, in combination with Pennington Biomedical Research Center. Survey questions were established based upon prevailing literature [8,16,19-24], professional dietetic field experience, and app design considerations. Athletes and sport professionals completed a web-based survey on their preferences for nutrition educational content, modes of in-app information and communication delivery, motivations and barriers that influence meal choices, and opinions on novel app functions. Participants in this study represent the primary end users for the Eat2Win app: athletes and sport professionals. Recruitment occurred through social media platforms, including Facebook, Twitter, and Instagram, along with targeted email campaigns using listservs housed at both My Sports Dietitian and Pennington Biomedical Research Center. The survey was open for 3 months, between May 4 and August 2, 2022. Survey data were collected and managed using Research Electronic Data Capture (REDCap) tools hosted at Pennington Biomedical [25,26]. REDCap is a secure, web-based software platform designed to support data capture for research studies, providing (1) an intuitive interface for validated data capture; (2) audit trails for tracking data manipulation and export procedures; (3) automated export procedures for seamless data downloads to common statistical packages; and (4) procedures for data integration and interoperability with external sources.

### Ethical Considerations

This research was reviewed and determined exempt by the institutional review board at Pennington Biomedical Research Center (IRB# 2021-045-PBRC). This was based on the following criteria: (1) research that only includes interactions involving survey procedures, and (2) the information obtained is recorded by the investigator in such a manner that the identity of the human subjects cannot readily be ascertained, directly or through identifiers linked to the subjects. A waiver of informed consent was approved, and an electronic consent to participate in research was provided to and acknowledged by participants before beginning the survey. Compensation was provided to participants that completed the survey by entry into a raffle and delivered through gift cards.

## Results

Respondent characteristics are presented in Table 1. A total of 1352 individuals responded to the survey as either an “athlete” or a “professional that works with athletes.” Respondents resided in over 6 countries but were primarily from the United States (1170/1306, 89.6%). Respondents from both sexes were well represented with 533 females and 773 males. Racial representation was similar to the greater United States population according to 2021 estimates of the United States Census Bureau [27] with the exceptions of a comparatively higher proportion of American Indians or Alaska Natives (118/1313, 9%) and a lower proportion of African Americans (103/1313, 7.8%). Respondents were also well educated, with 91.1% (1177/1292) reporting at least some college education. Broad representation was achieved across the level of athletics, years of participation in sport, and the primary sport from both athlete participants and sport professional area of practice; these data are presented in Table 2.

**Table 1:** Respondent characteristics. Participants were not excluded for individual missing data points.

		Athletes (N=441)	Professionals (N=911)
Age (years), mean (SD)		30 (10)	49 (5)
**Sex, n**			
	Male	256	517
	Female	156	377
	Nonbinary	2	1
	Prefer not to say	2	2
**Race, n**			
	American Indian or Alaska Native	30	88
	Asian	23	31
	Black or African American	43	60
	Native Hawaiian or Pacific Islander	14	11
	White	297	663
	Other	5	9
	Prefer not to say	3	11
	2 or more races	5	20
**Ethnicity, n**			
	Hispanic or Latino	140	293
	Not Hispanic or Latino	220	499
	Prefer not to say	28	46
	Unknown	22	33
**Education level, n**			
	GED^a^ or equivalent	12	9
	High school graduate	61	33
	Some college, no degree	111	99
	Associate degree	62	77
	Bachelor’s degree	108	284
	Master’s degree	37	322
	Doctorate degree	17	60
**Country, n**			
	Australia	15	9
	Canada	42	18
	European Union countries	3	10
	Mexico	7	5
	United States	336	834
	Other	9	18
**Professional credentials^b^, n**			
	Athletic Trainer	N/A^c^	283
	Registered dietitian or diet technician registered	N/A	316
	Doctor of Medicine or Doctor of Osteopathic Medicine	N/A	65
	Doctor of Physical Therapy	N/A	34
	Other	N/A	72
	No professional school degree	N/A	80

^a^GED: General Educational Development Test, educational certificate considered equivalent to a high school diploma.

^b^Athletes were not asked about professional credentials.

^c^N/A: not applicable.

**Table 2:** Sport or area of practice. Participants were not excluded for individual missing data points. Athletes were asked to answer regarding their participation in sports while sport professionals were asked to answer regarding the primary sport of athletes supported by their professional practice or the primary sport in which the athletes that they work with participate.

			Athletes, n		Sport professionals, n
**Level of athletics**					
	Youth	41		85	
	High school	69		252	
	NAIA^a^	14		29	
	NCAA^b^ Division 1	84		129	
	NCAA Division 2	60		80	
	NCAA Division 3	35		61	
	Professional	39		86	
	Club	77		165	
**Length of time in athletics or professional practice (years)**					
	<1	23		38	
	1-2	77		120	
	3-5	186		288	
	6-10	78		216	
	11-15	26		80	
	16-20	10		55	
	>21	18		94	
**Primary sport**					
	Baseball	27		36	
	Basketball	59		150	
	Bowling	7		6	
	Cheerleading	10		10	
	Cross country	13		23	
	Football	20		93	
	Golf	21		20	
	Lacrosse	14		13	
	Soccer	19		41	
	Softball	8		18	
	Swimming	49		42	
	Tennis	18		29	
	Track and field	69		66	
	Volleyball	16		38	
	Wrestling	6		17	
	Multisport athlete	34		185	
	Other	29		95	

^a^NAIA: National Association of Intercollegiate Athletics.

^b^NCAA: National Collegiate Athletic Association.

**Figure 1:**
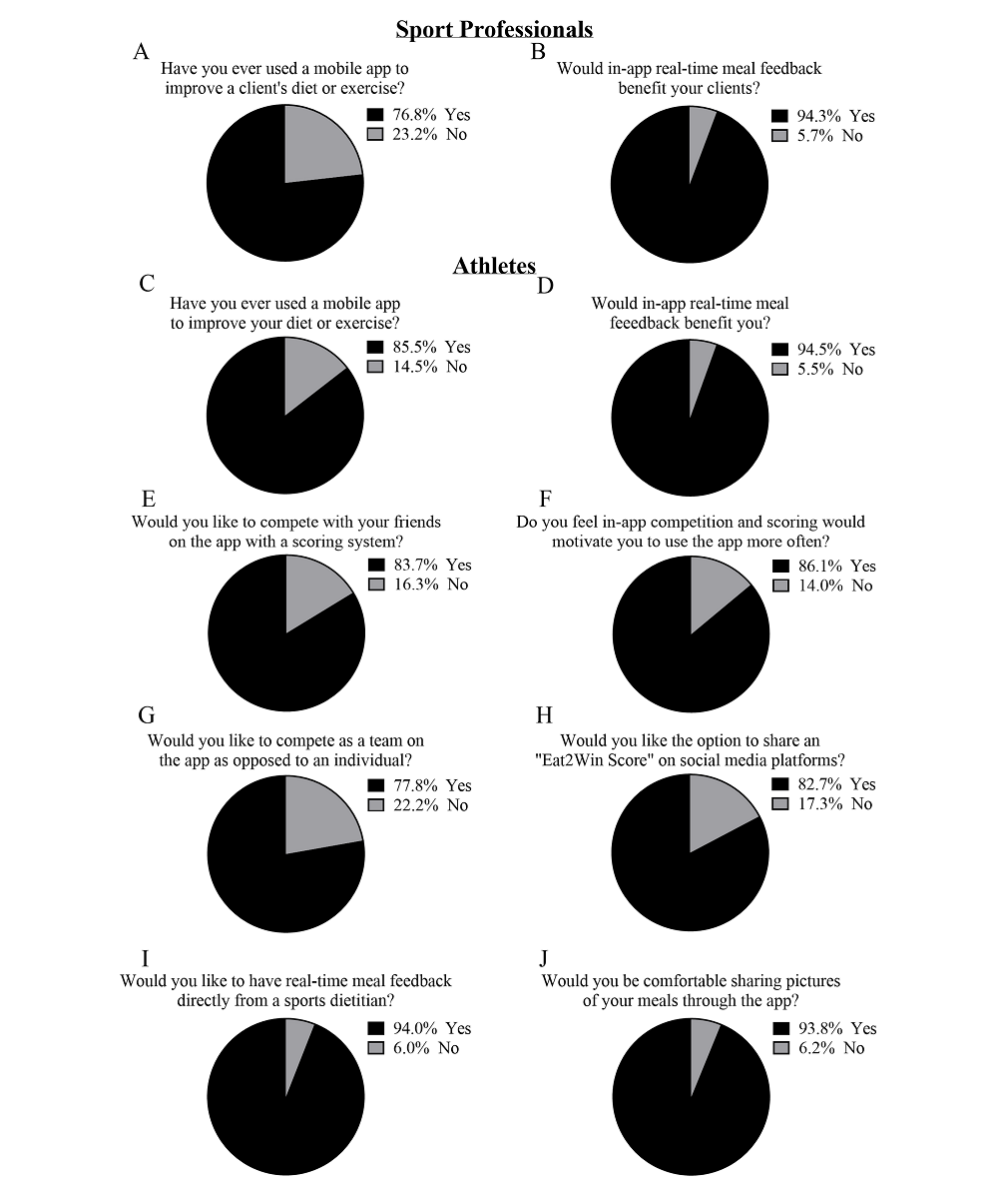
Previous mobile app use and interest in Eat2Win functions. Survey respondents were asked yes or no questions about their use of mobile apps and several Eat2Win functions. Separate questions were asked to athletes and sport professionals. Individual missing data points were excluded from analysis.

Respondent familiarity with the use of mobile apps for diet or exercise and respondent perception of the utility of select app functions are presented in Figure 1. A large majority (342/400, 85.5%) of athlete respondents reported using an app to improve their diet or exercise, while 76.8% (639/832) of sport professionals reported using an app to improve their client’s diet or exercise. Both athletes (380/402, 94.5%) and sport professionals (764/810, 94.3%) reported a perceived benefit to in-app real-time meal feedback. Various forms of in-app competition for athletes were positively viewed by 77.8%-86.1% of athlete respondents. Finally, athletes reported a desire for real-time meal feedback from a sports dietitian (380/402, 94.5%), and would be comfortable sharing pictures of their meals through the app (367/384, 95.6%).

**Figure 2:**
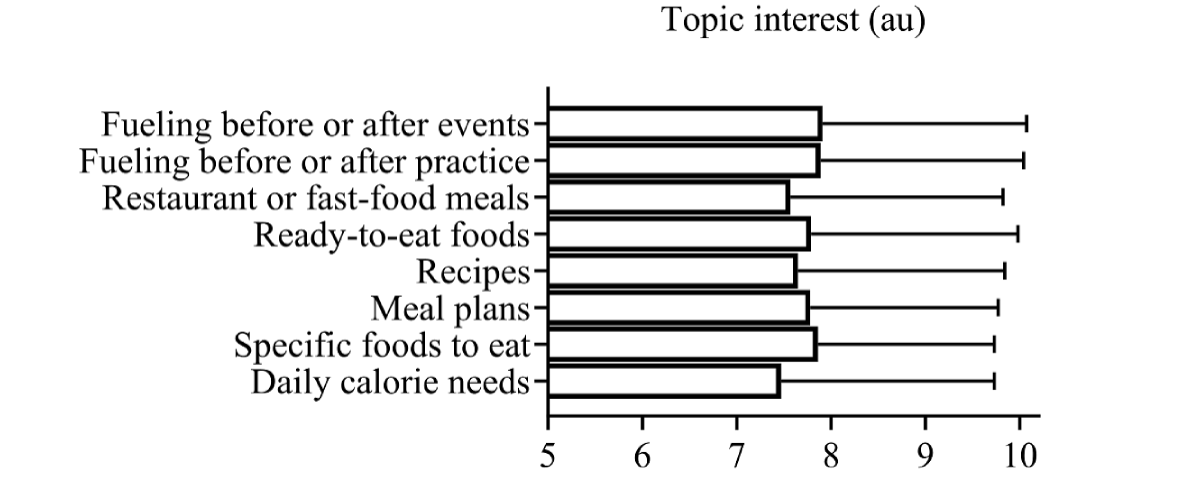
Interest in sports nutrition topics. Survey respondents were asked to rate their interest in a variety of sports nutrition topics, with 0 being not interested at all and 10 being extremely interested. Individual missing data points were excluded from analysis. Data are presented as mean (SD), n. Question (Q) 1: 7.9 (2.2), 1197. Q2: 7.9 (2.2), 1195. Q3: 7.6 (2.3), 1192. Q4: 7.8 (2.2), 1196. Q5: 7.7 (2.2), 1201. Q6: 7.8 (2), 1200. Q7: 7.9 (1.9), 1205. Q8: 7.5 (2.3), 1201. Au: arbitrary units.

**Figure 3:**
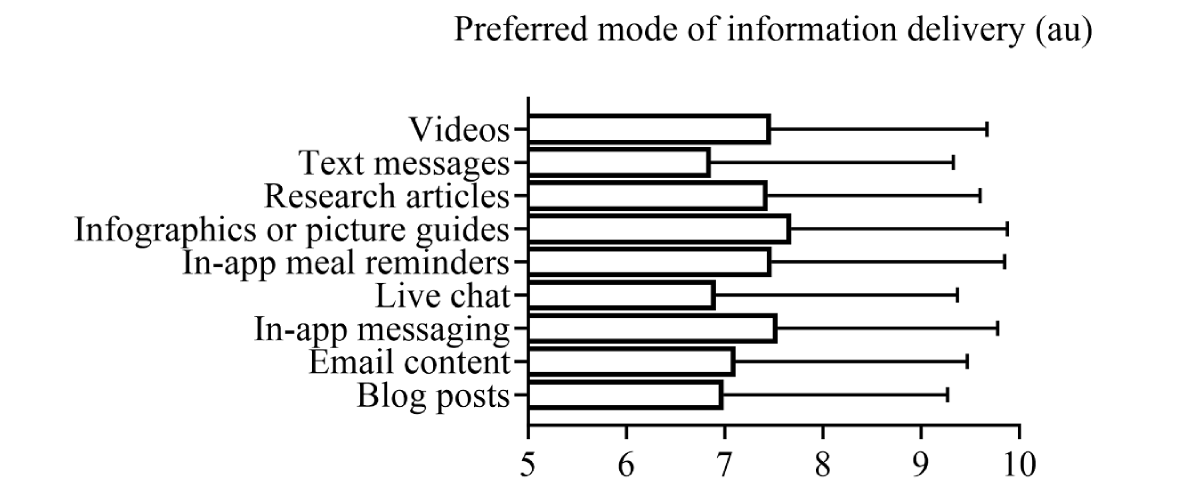
Preferred mode of information delivery. Survey respondents were asked to rate their preferred mode of delivery for information on sports nutrition topics, with 0 being not interested at all and 10 being extremely interested. Individual missing data points were excluded from analysis. Data are presented as mean (SD), n. Question (Q) 1: 7.5 (2.2), 1191. Q2: 6.9 (2.5), 1191. Q3: 7.4 (2.2), 1193. Q4: 7.7 (2.2), 1196. Q5: 7.5 (2.4), 1186. Q6: 6.9 (2.5), 1193. Q7: 7.5 (2.2), 1188. Q8: 7.1 (2.4), 1193. Q9: 7.0 (2.3), 1193. Au: arbitrary units.

All respondents were asked to rank their interest in 8 core sports nutrition topics from 0 to 10, with 10 being extremely interested and 0 being not interested at all. Results for the 8 core sports nutrition topics are presented in Figure 2. All topics were of high interest, with a rating between 7.5 and 7.9 au, with “fueling before or after events and practices” receiving the highest interest ratings. Respondents were then asked to provide their interest in the format in which they would receive information on sports nutrition topics. Interest in 9 modes of information delivery is presented in Figure 3. Visual formats (video 7.5 au and infographics 7.7 au) and in-app alerts (direct messaging 7.5 au and meal reminders or notifications 7.5 au) received the highest interest ratings.

**Figure 4:**
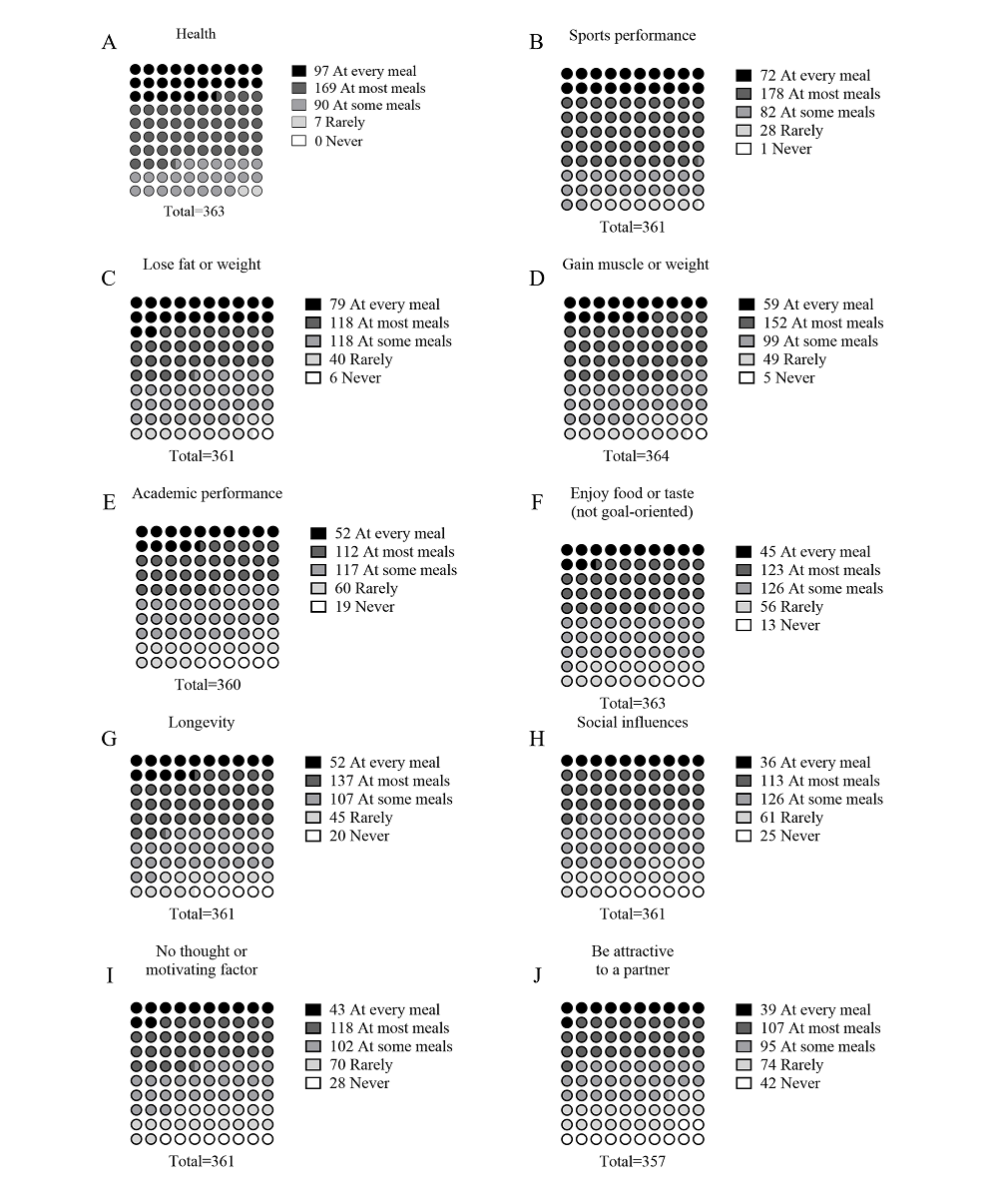
Motivators to meal choices. Athlete respondents were asked about 10 motivators to meal choices and the frequency of which those motivators influenced meal choice using a modified Likert scale. Frequency data are presented (number of respondents that selected each item).

**Figure 5:**
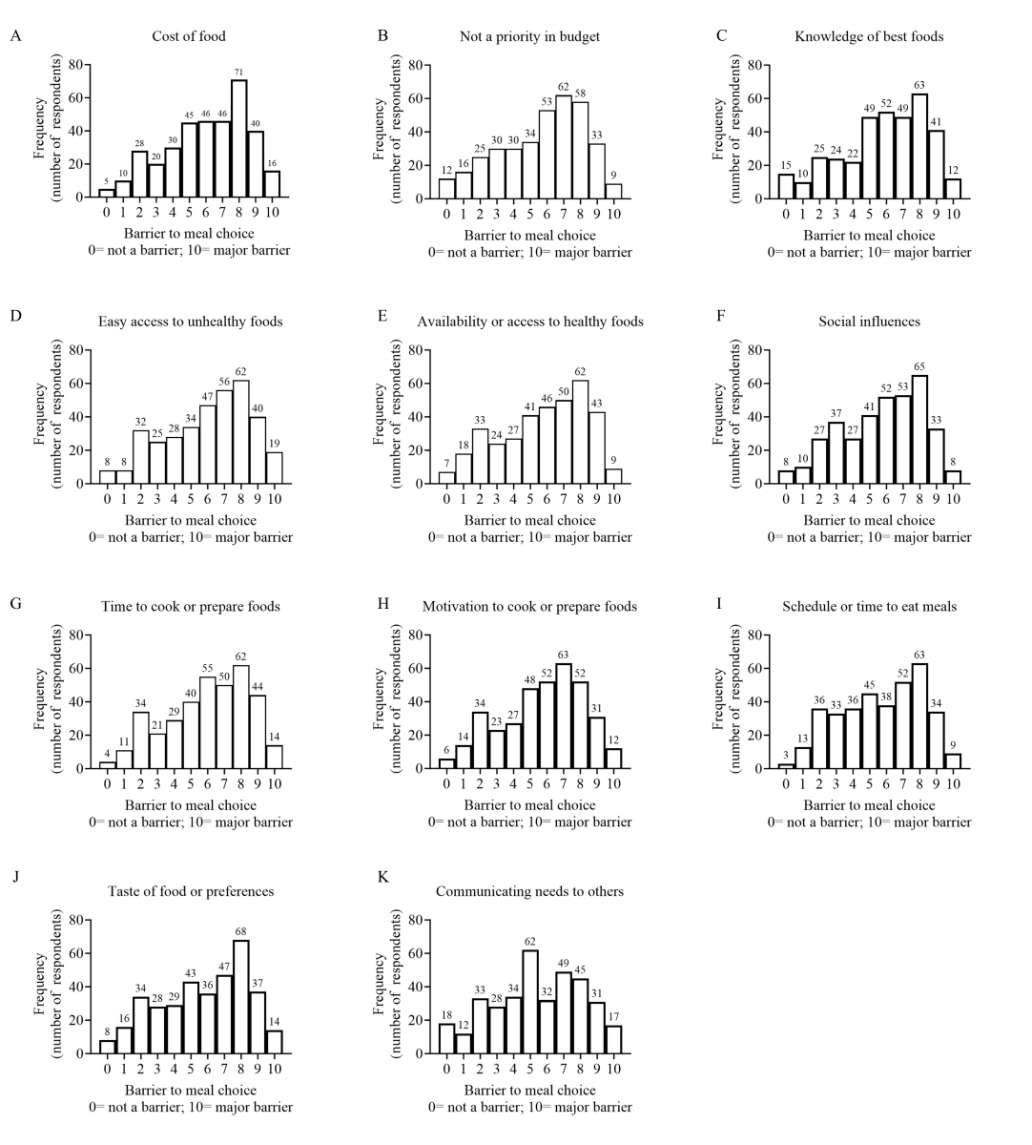
Barriers to meal choices. Athlete respondents were asked about 11 common barriers that influenced their meal choices and to rate those barriers on a 0-10 scale, with 0 representing not a barrier at all and 10 signifying a major barrier. Frequency data are presented (number of respondents that selected each item).

Only the athlete respondents were asked additional questions about their motivators and barriers to exercise. The athlete respondents were asked about the frequency with which 10 motivating factors influenced their meal selection using a modified Likert scale. Data on whether each motivating factor influenced meal choices for the following categories: (1) at every meal, (2) at most meals, (3) at some meals, (4) rarely, or (5) never (Figure 4). Respondents reported that 2 motivating factors influenced food choices most often: “health” and “sports performance.” “Health” influenced meal choices at every meal or at most meals in 73.3% (266/363) of athlete respondents, while “sports performance” was reported at every meal or at most meals in 69.3% (250/361) of athlete respondents. The 2 motivating factors with the least frequency were “attracting a partner,” influencing meal choices rarely or never in 32.5% (116/357) of athlete respondents, or “no motivating factor or not thinking about food choice,” influencing meal choices rarely or never in 27.2% (98/361) of athlete respondents. The athlete respondents were also asked to rate 11 common barriers that may influence meal choices using a 0-10 scale, with 0 representing not a barrier at all and 10 signifying a major barrier. Data on how athletes rated each barrier are presented in Figure 5. The highest rated barriers were the cost of food (Barrier Rating: mean 6, SD 2.5 au; n=357), easy access to unhealthy food (mean 6, SD 2.6 au; n=359) and time to cook or prepare food (mean 6, SD 2.5 au; n=364), while the lowest rated barrier was communication with others (mean 5.4, SD 2.7 au; n=361).

## Discussion

### Overview

This formative research study describes the motivators and barriers to nutritional intake of athletes, along with the preferences and perceptions of sports nutrition content and mobile app functions of both athletes and sport professionals. These 2 populations represent the primary end users for the novel athlete-nutrition mobile app, Eat2Win. The Eat2Win app aims to improve athlete nutritional intake as a means to combat the underrecognized, yet highly damaging, malnutrition and RED-S observed in this population [28]. This is important to address because athlete malnutrition and RED-S, including symptoms and preventative nutrition solutions, are not effectively addressed by athletes or sport professionals (eg, coaches and athletic trainers). One recent report suggests a majority (87%) of competitive athletes aged 14-34 years present with at least one health-related symptom of RED-S [29]. Although athletes are typically considered a healthy population, there is a clear gap in nutrition knowledge and nutrition oversight in this population that leads to the high prevalence of RED-S. This gap is due in part to barriers that prevent access to evidence-based nutrition information and experts in sports nutrition. Mobile apps aimed at athletes and sport professionals represent an opportunity to address the critical barriers to optimal athlete nutritional intake and prevent RED-S, including risk assessment and nutrition education for athletes and individuals that work with athletes.

Uniquely, the Eat2Win app connects athletes and sport professionals directly with expert sports dietitians, along with providing in-app functions such as sports nutrition education, nutritional intake monitoring, and personalized feedback on food and meal choices that may help combat the risk of athlete malnutrition. Notably, athletes report using coaches and athletic trainers as their primary sources of sports nutrition information [8,30], thus it is imperative to simultaneously improve the sports nutrition knowledge of athletes and sport professionals concomitantly. These concepts align with established effectiveness of in-person approaches [10-12,14]. Our survey data of athletes and sport professionals describe a high interest in sports nutrition topics and have provided information regarding preferred modes of in-app education delivery along with the motivators and barriers that influence meal choices. This information will be used to further develop Eat2Win app content to better provide the requisite sports nutrition knowledge and access to a sports dietitian to end users (athletes and sport professionals).

Parts of this survey were also used to better understand the perceptions of the unique app functions by potential end users. More survey respondents reported a perceived benefit to receiving real-time meal feedback and connecting with a sports dietitian compared to functions of in-app competition, either as individuals or as teams. Regarding the benefit to real-time meal feedback, which would require in-app food photography submitted by the app users, we report a high acceptability to submitting photos of foods and meals by the survey respondents. This acceptability is important, as one of the next steps in app development is to generate app-based algorithms to predict RED-S risk in athletes and to optimize the scalability of the app by delivering automated (artificial intelligence–generated) real-time feedback to users at mealtime, using in-app food photography submitted by app users. We anticipate the next iteration of app development will place an emphasis on the highest priority items reported by the survey respondents, including real-time meal feedback, access to sports dietitians, and video and infographic educational content delivery.

These survey results should be interpreted with several limitations in mind. We did not assess whether athletes were in their sport’s in-season or off-season when completing this survey. Others have proposed a benefit to delivering educational curriculum during specific training periods [31]. Whether respondent interest in Eat2Win app content and functionality changes over the course of a sport’s in-season and off-season cycle is unknown. We plan to address this knowledge gap in future trials using the Eat2Win app by comparing app use between in-season and off-season conditions. Although respondents completed the survey from more than 6 countries, the vast majority were from the United States; thus, we cannot assess whether we sampled representative populations from other countries due to the small sample size of respondents outside of the United States. Additionally, the survey respondents were representative of the general population demographics of the United States, with a broad geographic spread. Two states were not represented in the survey (Rhode Island and Vermont). It is also possible that the population that completed this survey represent individuals that are specifically conscious of or educated about nutrition compared to the average athlete or end user of the app. The survey was advertised broadly on social media, but it was only available in English, which may have prevented additional representation from other countries or from non–English-speaking residents of the United States. This limits our ability to speak beyond the representative, English-speaking population in the United States. Despite these limitations, this formative research provides meaningful data on athletes and sport professionals and will aid in the continued development of the Eat2Win app to target athlete nutrition and the prevention of RED-S.

### Conclusions

The Eat2Win app is designed to improve nutritional intake in athletes by empowering athletes and sport professionals with evidence-based nutrition information, real-time recommendations on meal choices, and direct exposure to expert sports dietitians. This formative research was conducted on the Eat2Win app’s primary end users and details the preferences for nutrition education content, modes of in-app information and communication delivery, motivations and barriers that influence meal choices, and opinions on in-app functions. This data will inform the modifications that will be implemented in future iterations of the app. Continued research using the Eat2Win app will determine whether the platform can improve athlete nutrition and reduce the risk of RED-S and malnutrition in athletes.

## Appendix

Multimedia Appendix 1 Table S1. Meal feedback template statements.

## Abbreviations

REDCap: Research Electronic Data Capture
RED-S: relative energy deficiency in sport

## Data Availability

The data sets generated and analyzed during this study are available from the corresponding author on reasonable request and in alignment with the institutional policies at Pennington Biomedical Research Center.
