# Supplementary material for: Factors Influencing Nutritional Intake and Interests in Educational Content of Athletes and Sport Professionals Toward the Development of a Clinician-Supported Mobile App to Combat Relative Energy Deficiency in Sport: Formative Research and a Description of App Functions
Source: JMIR Form Res. 2023 Jul 26;7:e45098. doi: 10.2196/45098 (PMC10413240; doi:10.2196/45098)
Supplement: Multimedia Appendix 1 [file formative_v7i1e45098_app1.docx]

**Table S1. Meal Feedback Template Statements**

| - Increase Quality Protein Intake - Maintain Quality Protein Intake - Reduce Protein Intake - Add Fruit to your Meal - Add Vegetables to your Meal - Increase Quality Carbohydrate Intake - Maintain Quality Carbohydrate Intake - Reduce Carbohydrate Intake - Increase Healthy Fat Intake - Maintain Healthy Fat Intake - Decrease Fat Intake - Limit Fried Foods - Limit Processed Foods - Reduce High-Calorie Drink - Add a High-Calorie Drink to your Meal - Add Post-Workout Calories - Add Post-Workout Carbohydrates - Add Post-Workout Protein - Increase Overall Caloric Intake - Decrease Overall Caloric Intake - Great Job! Keep it Up! - You Made Great Choices! |
| --- |
